# Psychometric Analysis of an Adapted Patient Care Ownership Scale for Medical Students

**DOI:** 10.5334/pme.1820

**Published:** 2025-11-24

**Authors:** Troy K. Kincaid, Wendy Christensen, Jennifer E. Adams, Tai Lockspeiser, Michele Kiger

**Affiliations:** 1 University of Colorado School of Medicine, Aurora, Colorado, US; 2 Uniformed Services University, Bethesda, Maryland, US; 3 Center for Education Research and Scholarship, University of Colorado US

## Abstract

**Purpose:**

Patient care ownership (PCO) is a commitment to patient care with important implications for both patients and providers, and understanding PCO among trainees is an emerging area of study. Recently, Djulbegovic et al adapted a psychological ownership scale for graduate medical education (GME). Tailoring this scale for undergraduate medical education (UME) would strengthen the ability to measure and promote PCO among students, while directly linking this growth to the transition to GME.

**Method:**

Djulbegovic et al.’s PCO scale was adjusted through content expert input and cognitive interviews. This scale was administered to post-clerkship students at the University of Colorado School of Medicine after academic years 2020–21 and 2021–22. Exploratory factor analysis (EFA) was used to examine the underlying themes of the adapted scale in the 2020–21 sample. Confirmatory factor analysis (CFA) was performed in the 2021–22 sample to evaluate factors elucidated in EFA. Messick’s validity framework was used to guide collection of content, response process, and internal structure validity evidence.

**Results:**

The final scale included 16, 7-point Likert-style items. EFA modeling in the first sample suggested either a four-factor structure or a two-factor structure that was a simplification of the four-factor structure. CFA modeling in the second sample supported a four-factor model of PCO in medical students, named Advocacy, Decision-making, Opportunity, and Responsibility.

**Conclusions:**

This PCO scale demonstrated strong internal structure validity evidence and identified four factors contributing to PCO in medical students. Comparing these to Djulbegovic’s work elucidates differences between UME and GME learners’ experiences of PCO, chiefly in the opportunity of care ownership.

Patient care necessitates complex, interrelated decisions in the context of invested relationships between patients and care providers. Patient care ownership (PCO) is a term that encompasses the commitment between the provider and the patient [1,2]. Although psychological ownership has been studied for decades in the fields of cognitive and organizational psychology [3], PCO has more recently become an emerging area of research in medical education. The literature has defined PCO as the affective commitment of a medical provider toward patients [4], which entails both internal sentiments, such as autonomy and self-efficacy, and practice-based tasks such as clinical follow-up, care coordination, and advocacy [2,5]. Whereas most prior studies have focused on PCO within the context of graduate medical education (GME) [6,7,8,9,10], the development of PCO in medical students has also emerged as an important area of study, as care responsibility is one of the defining features of the transition to residency. Further, the changing undergraduate medical education (UME) landscape, with condensing preclinical learning and evolving clinical curricular modalities (i.e., increasing numbers of longitudinal integrated clerkships (LICs)), could impact how PCO is fostered in medical students [11].

To address this question, we need tools to measure PCO. Djulbegovic et al. first adapted a psychological ownership survey developed by Avey and colleagues [12] for use with GME trainees [13]. The Avey scale was created to identify ownership in business organizations and was adapted by Djulbegovic et al. to apply to a medical training patient-care setting. Subsequently, Wyatt and colleagues revised Avey’s original scale to create a scale for medical students independent of Djulbegovic’s adaptations, citing the inaccessibility of resident tasks in Djulbegovic’s scale to medical students [14]. Accordingly, the Wyatt scale measures PCO with cognitive-focused items in line with Avey’s scale (e.g., “I feel/I enjoy / I am / I have a right to”) and is distinct from the Djulbegovic scale, which focuses more on behaviors. However, some resident tasks outlined in the Djulbegovic scale might be accessible to students in the correct clinical setting. As such, a tool that focuses more on student-level behaviors tied to PCO is still needed. Additionally, Wyatt et al.’s tool sought validity evidence in a traditional block rotation setting. With LICs becoming more popular, a scale with validity evidence for students across a variety of clinical clerkship models would hold additional value, particularly as such rotations such as LICs or advanced clinical rotations (i.e., acting internships or sub-internships) might allow for increased degrees of autonomy that were not envisioned during the development of Wyatt’s scale. As such, existing tools do not encompass the full scope of PCO available to medical students in some settings, which is important to capture as educators seek to foster the longitudinal development of PCO across the continuum from UME to GME.

Therefore, we adapted a scale for measuring PCO using a different theoretical approach to development than that of Wyatt et al. and studied its psychometric properties for use in UME. Specifically, we approached this revision with the assumption that students would be allowed greater autonomy in practice over time as they move closer to the GME transition making PCO a critical element of professional development for UME trainees. Our conceptual framework drew on social-cognitive theories [15], which posit that learning is dependent on the social environment in which it occurs. Specifically, we drew upon legitimate peripheral participation [16], which describes how newcomers are welcomed into a community of practice through authentic immersion and adopt more central roles within the community over time. We believe this framework is apt for the study of PCO because students must adopt progressively central roles in patient care over time, which includes behaviors associated with PCO. In addition, prior studies have suggested that the development of PCO among students involves a socialization process in which they adopt attitudes and behaviors more central to the role of physician over time which is in line with the tenets of legitimate peripheral participation [17]. For example, a team on the medical wards could represent a community of practice wherein ownership over a patient’s care is felt among team members, and responsibility and decision making for each aspect of this patient’s care (e.g., presentation on rounds, writing orders, and organizing home health needs) is shared among team members (e.g., medical student, intern, and supervising resident). As junior trainees demonstrate increasing levels of medical knowledge and assume greater levels of responsibility, they progress to occupying more central roles on the team both in cognitive and task ownership. As Lave and Wenger describe, this cognitive apprenticeship [16] creates new and meaningful learning.

With these theoretical underpinnings, we adapted the Djulbegovic PCO scale (developed for GME learners) to a population of medical students in their core clerkship year using best practices for evidence-based survey development [18]. Aligned with our conceptual framework, which presumes medical students can, through legitimate peripheral participation, learn to perform the type of PCO tasks on the scale designed for GME trainees, we adapted our scale for medical students from the Djulbegovic scale. We collected validity evidence for this new scale using Messick’s validity framework, seeking to describe the psychometric properties of a patient care ownership scale for medical students adapted directly from a PCO scale originally designed for GME [19].

## Method

### Study Setting and Participants

The University of Colorado, School of Medicine (CUSOM) enrolls approximately 184 medical students each year. During the study period, the principal clerkship year takes place during the third academic year, with students enrolled in either traditional block rotations (TBRs) or longitudinal integrated clerkships (LICs). Students in the LIC curriculum participate in comprehensive care of patients over time, engage in continuity relationships with clinical faculty, and meet the core clinical competencies across all traditional disciplines simultaneously during their core clinical year [20]. Rather than traditional block clerkships organized into a series of discipline-specific blocks, LICs proceed in a parallel, non-sequential fashion. For example, internal medicine, surgical, pediatric, obstetric, psychiatric, and emergency experiences are interspersed as half-days throughout the entire primary clinical year rather than discrete month-long blocks. The study was approved by the Colorado Multiple Institution Review Board (#22-0198). Participant data was anonymized by non-author staff members of the Office of Assessment, Evaluation, and Outcomes and analyzed in aggregate.

### Scale Adaptation

We adapted Djulbegovic, et al.’s 15-item PCO scale using Messick’s validity framework as a guide for collecting validity evidence for our revised scale [19,21]. The Djulbegovic scale was designed to explore eight dimensions of PCO and identified three potential subscales through exploratory factor analyses (EFA). These were named assertiveness, being the “go-to” person, and diligence. In this paper, we focus on gathering evidence for content, response process, and internal structure validity for this new scale. To build content validity, we (TK, TL, MK, JEA) considered prior literature on PCO to independently propose revisions to scale questions, then collaboratively agreed upon in-line adaptations to the PCO scale. Items were modified to apply to UME learners or removed when not applicable to students. Additionally, we asked two experts in LIC development and assessment as well as two national experts in LICs and PCO to review and provide feedback on the revised 15-item survey. Small syntactical changes were also made to the adapted scale at this stage. Next, to gather evidence of response process validity, we conducted cognitive interviews with eight fourth-year medical students during academic year 2020–21 (AY 21), who were purposefully sampled to represent those who completed TBR and LIC clerkships (four students from each). Cognitive interviews were conducted by one author (TK) using a think-aloud approach, followed by scripted verbal probing that included specific questions about whether or not each item was achievable for medical students (Appendix A) [18]. At this stage, two items were added to make a 17-item survey. One of these was a new item entirely. The other divided a single question about whether students felt comfortable advocating for medical or social needs of their patients into two separate items. Finally, the authors revised items according to qualitative themes derived from all collected feedback to arrive at the final adapted scale.

### Data Collection

The adapted scale was incorporated into a required end-of-year survey designed to gather information from the students about the curriculum and the medical school in general. Although students are required to take the survey, responses to specific items or scales are optional. Medical students were asked to complete the survey via email and were incentivized with $5 Amazon gift cards upon completing the entire survey. For this study, we utilized responses from 3^rd^ and 4^th^ year medical students at CUSOM (EOY3 and EOY4) during the academic years 2020–21 and 2021–22 (AYs 21 and 22) in Qualtrics™ (Seattle, WA).

### Data Analysis

To empirically identify plausible internal structures of the PCO scale and assess evidence of the internal validity of those structures in a subsequent sample, we conducted exploratory and confirmatory factor analyses. All analyses were performed in SAS™ 9.4; the EFA and confirmatory factor analyses (CFA) were conducted using the FACTOR and the CALIS procedures, respectively [22].

The first round of analysis was conducted using data from the AY 21 third-year student cohort (the first group of students to receive the survey incorporating the adapted PCO scale). This analysis used EFA to explore the psychometric properties of the scale in its first administration. After data cleaning, EFA proceeds through a five-step process: extraction, deciding the number of factors to retain, rotation, interpretation (with possible return to earlier steps), and replication [23]. We used principal axes factoring because it is consistent with the common factor model and does not have distributional assumptions [24]. To determine the number of factors to extract, the eigenvalues from an initial (i.e., non-rotated) extraction were evaluated using the following criteria in conjunction: scree plot examination, the Kaiser criterion (eigenvalues > 1), the minimum eigenvalue criterion (eigenvalues > average of eigenvalues), and the proportion of variance criterion (minimum number of factors whose cumulative proportion of shared variance was at or above 100%). Once the number of factors to extract was decided, promax rotation – an oblique rotation that permits factor correlations, as anticipated – was used to clarify the final factor loadings. The best factor solutions are those with simple structure, in which each item loads strongly onto just one factor and weakly on all others. The communalities, the factor correlations, pattern matrix of factor loadings, and structure matrix of item-factor correlations for each number of extracted factors were examined to decide which factor solutions to choose.

The second round of analysis was conducted using data from AY 22, collected at the end of the year for third- and fourth-year students, many of whom in the latter group had participated in the administration of the survey in the previous year. The focus of the second round of analysis was to see if the factor solutions chosen in the first round replicated in a new sample. CFA models were fitted according to the chosen factor models and compared to a one-factor confirmatory model using likelihood ratio tests. Because many of the fourth-year students in the AY 22 sample were part of the third-year sample used in the first round of analysis, CFA models were fitted separately to the third- and fourth-year students in the second sample to examine differences between cohorts. Before fitting the CFA models, we verified that each model was over-identified – that is, the number of observed variances and covariances exceeded the number of estimated parameters – to ensure that model estimation and model comparisons were possible [25]. All CFA models we tested were over-identified, making them both estimable and suitable for evaluating relative model fit. The authors independently named scale factors before group reconciliation, and group consensus on names was informed by a literature review of PCO and psychological task ownership.

Kline recommended using coefficient omega (also known as composite reliability) instead of Cronbach’s alpha to assess within-factor reliability in CFA models because it accounts for items on the same factor having different factor loadings and error variances [25]. The omegas for the Responsibility factor were 0.84 (EOY 3) and 0.87 (EOY 4); for Advocacy, 0.80 and 0.89; and for Opportunity, 0.73 and 0.75. These were all higher than the omegas from the Decision-making factor (EOY 3: 0.49, EOY 4: 0.65). Notably, the reliability of the Decision-making factor was higher in EOY 4 than in EOY 3, such that its reliability in EOY 4 was closer to that of the other factors.

Kline also recommended examining the average variance extracted (AVE) within factors to assess convergent validity [25]. The AVEs for the EOY 3 model were: Responsibility (0.46), Advocacy (0.52), Opportunity (0.67), and Decision-making (0.35). The AVEs for EOY 4 were: Responsibility (0.50), Advocacy (0.67), Opportunity (0.52), and Decision-making (0.50). In the EOY 4 model, Decision-making and Advocacy had higher AVEs, but the AVE for Opportunity was higher in the EOY 3 model.

## Results

### Scale Adaptation

The scale administered to students included 17 items; two of which were additions to Djulbegovic et al.’s scale. Appendix B summarizes justifications for adaptations to the Djulbegovic scale. These additional items were added after cognitive interviews with medical students and input from the faculty experts. The first new item divided a single question that lumped medical and social care responsibilities in the original scale into two distinct items, as this was identified as a pertinent delineation for medical students. The second new item was added to directly interrogate student’s opportunity for care ownership. The same 7-point Likert-type scale used by Djubegovic et al was used for this scale.

### First-round analysis

A total of 183 were invited to participate in the AY 21 EOY 3 survey, which included the PCO scale among other measures. Students were excluded from this analysis if they did not respond to any PCO items, skipped multiple PCO items (two students, both skipping six of the 17 items), selected “neither agree nor disagree” for all PCO items (two students), or were a member of the research team (one student). No students skipped just one item. This resulted in an analytic sample of 176 (LIC = 44, response rate: 96.2%), all of whom answered all PCO items. Item means ranged from 4.1 to 5.8, and the inter-item correlations ranged from 0.16 to 0.80. The skewness of the items ranged from –1.24 to –0.02, and kurtosis ranged from –1.00 to 3.00. Appendix C lists item means and standard deviations. The Cronbach’s alpha was high at 0.92.

EFA was used to identify the underlying factor structures of PCO as measured in our scale. First, an unrotated eight-factor model, matching the theorized number of factors for the Djulbegovic scale, was applied, and the eigenvalues were examined to determine how many factors to extract for the EFA. Examination of the scree plot and the Kaiser criterion favored a two-factor solution, while the minimum eigenvalue criterion favored a three-factor solution, and the proportion of variance criterion favored a four-factor solution. Across all examined models, the inter-factor correlations ranged from 0.44 to 0.61, and most items correlated with multiple factors at 0.4 or above. We examined the factor loadings across the three models for evidence of cross-loadings, which occur when items load strongly onto more than one factor. Item 17 cross-loaded in both the two- and three-factor models, and Item 5 did so in the three-factor model. In contrast, all items in the four-factor model loaded strongly onto only one factor (i.e., the model exhibited simple structure).

To explore the degree to which Item 17 influenced the internal structure, we re-conducted the EFA for each model with Item 17 removed and found minimal changes to the factor structures and loadings. Given the stability of all other factor loadings regardless of Item 17’s inclusion, we proceeded with the EFA models that excluded Item 17.

In all three models, Items 6 through 13 loaded strongly onto a single factor. The remaining items – Items 1 through 5 and Items 13 through 16 – loaded together strongly onto the second factor in the two-factor model. The three- and four-factor models thus were distinguished by how they separated the items in that latter factor of the two-factor model. In both models, Items 1 through 4 loaded strongly together on one factor, as did Items 13, 14, and 16. In the three-factor model, Item 5 did not load strongly on any factor and also showed non-trivial cross-loadings (0.27 and 0.31) on the three specific factors. Item 15’s loading with Items 13, 14, and 16 was 0.43, which met our criterion of 0.4 but not by much. In the four-factor model, Items 5 and 15 loaded together strongly. Item 5 did not cross-load with any other factor, and Item 15 had the highest loading across all models (0.54). Because the four-factor model had a simple structure and removing Item 5 to resolve cross-loadings in the three-factor model would have eliminated the four-factor model from consideration, we proceeded with the two- and four-factor models in the subsequent confirmatory analysis. The factor loadings for the two- and four-factors models with Item 17 omitted are shown in Table 1.

**Table 1:** Pattern matrix exploratory factor loadings and factor names for the final two-factor and four-factor solutions in CUSOM students after AY 2020–2021.


	TWO-FACTOR SOLUTION		FOUR-FACTOR SOLUTION			
	
	RESPONSIBILITY	INITIATIVE	RESPONSIBILITY	ADVOCACY	OPPORTUNITY	DECISION-MAKING

Q1	0.20971	**0.52804**	0.18749	**0.7178**	–0.0435	–0.0761

Q2	0.0167	**0.84089**	–0.0135	**0.7866**	0.12457	0.06524

Q3	–0.0615	**0.84351**	–0.0957	**0.80439**	0.14002	0.03453

Q4	0.17729	**0.55284**	0.1453	**0.61876**	–0.1187	0.16906

Q5	0.00433	**0.52645**	–0.0418	0.21853	–0.045	**0.55661**

Q6	**0.60536**	–0.0213	**0.5904**	–0.0616	–0.1069	0.19789

Q7	**0.83103**	0.00623	**0.8181**	–0.0626	0.02969	0.07777

Q8	**0.73965**	–0.0245	**0.72713**	0.12033	–0.0875	–0.0538

Q9	**0.66371**	0.14417	**0.65607**	–0.0224	0.08486	0.1417

Q10	**0.62995**	0.17785	**0.61901**	0.13991	0.06222	0.01522

Q11	**0.68735**	0.05728	**0.68155**	–0.0064	0.0845	0.00335

Q12	**0.74984**	0.01384	**0.76029**	0.11594	0.06506	–0.1963

Q13	0.02913	**0.74181**	0.02798	0.0909	**0.55273**	0.25756

Q14	–0.0109	**0.57344**	–0.0057	–0.0193	**0.80774**	–0.1199

Q15	0.15091	**0.43162**	0.12745	–0.0242	0.0991	**0.53829**

Q16	0.0462	**0.81313**	0.03844	0.07304	**0.80469**	0.1134


Abbreviations: CUSOM = University of Colorado School of Medicine; AY = academic year. ^a^ Loadings above |0.4| are bolded. ^b^ The items included in each named factor (column) have a grey background.

**Figure 1:**
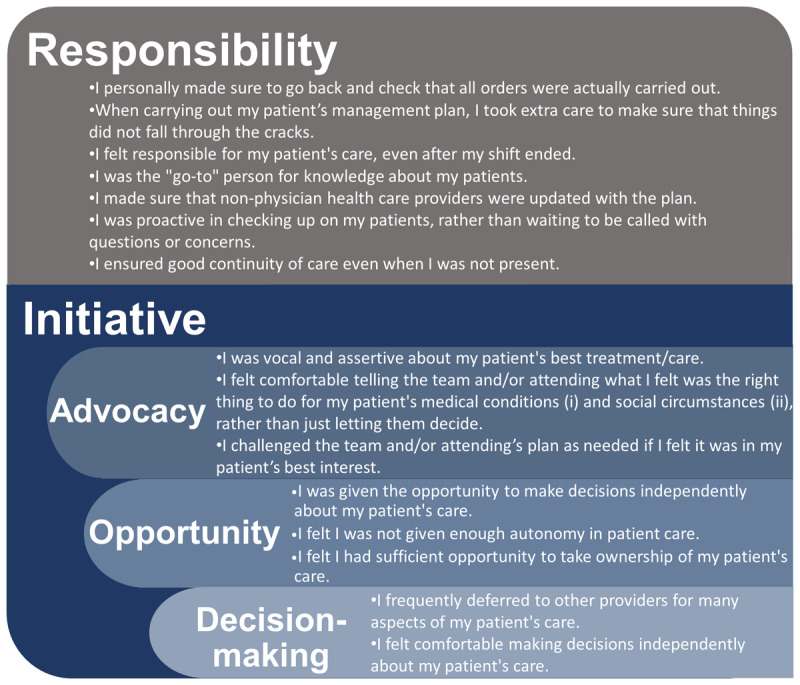
Patient care ownership scale items grouped according to factor analyses of a survey administered to CUSOM students after 3^rd^ and 4^th^ years of medical school during AYs 2020–21 and 2021–22. These are embedded within another hypothesized two-factor solution (Responsibility, Initiative) which had less robust evidence upon confirmatory analysis.

In the two-factor model, Items 6–12 formed a factor that we named “Responsibility” and Items 1–5 and 13–16 formed a factor that we named “Initiative”. The four-factor model also contained the Responsibility. The other three factors were “Advocacy” (Items 1–4), “Opportunity” (Items 13, 14, and 16), and “Decision-making” (Items 5 and 15). These are shown in Figure 1. Although factors with only two items are less desirable than factors with three or more items we chose to retain the Decision-making factor due to its strong loadings and theoretical significance as a component of PCO [25].

### Second-round analyses

A total of 180 students were invited to participate in the AY 22 EOY 3 survey. Students were excluded if they did not respond to any PCO items or selected “neither agree nor disagree” for all PCO items (three students), resulting in an analytic sample of 167 (LIC = 45, response rate: 92.8%). Item means ranged from 4.7 to 6.8, and the inter-item correlations ranged from –0.01 to 0.73. The skewness of the items ranged from –1.70 to –0.19, and kurtosis ranged from –1.17 to 3.83. Cronbach’s alpha was 0.86. A total of 182 students were invited to participate in the AY 22 EOY 4 survey. Students were excluded for not responding to any PCO items or being a member of the research team (one student), resulting in an analytic sample of 151 (LIC = 38, response rate: 85.2%). Item means ranged from 5.01 to 6.64, and the inter-item correlations ranged from 0.14 to 0.86. Appendix C lists item means and standard deviations; the skewness ranged from –1.65 to –0.36, and the kurtosis ranged from –0.91 to 2.67. Cronbach’s alpha was 0.91.

CFA was first used to determine whether the two- or four-factor solutions identified in the first-round analysis EFA fit best. Although the four-factor model included a two-item factor, we confirmed that it was over-identified by directly computing the degrees of freedom. In addition, the model satisfied the “two-indicator rule” described by Kline, a commonly used heuristic for assessing model identification in CFA [25]. For the EOY 3 and EOY 4 samples separately, three CFA models were fitted: a one-factor model, a two-factor model with the Responsibility and Initiative factors specified, and a four-factor model with the Responsibility, Advocacy, Opportunity, and Decision-making factors specified. For both samples, the likelihood ratio test indicated that the two-factor model fit better than the one-factor model (EOY 3 p < .001; EOY 4 p < .001), and the four-factor model fit better than the two-factor model (EOY 3 p < .001; EOY 4 p < .001. All factor loadings were positive and significantly different from zero (all p < 0.001), and all factors correlated significantly (all p < .01). The unstandardized factor loadings are shown in Table 2.

**Table 2:** Unstandardized factor loadings for four-factor CFA model across two data sets: CUSOM EOY 3 and 4 PCO respondents after AY 2020–21 and 2021–22. Factors hypotheses based on four-factor EFA solution demonstrated in Table 1.


FACTOR NAME	ITEM	EOY 3		EOY 4	
	
		LOADING	SE	LOADING	SE

Responsibility	06	1.08885	0.14671	1.13732	0.12858

	07	0.91752	0.12335	0.89133	0.11778

	08	1.17404	0.16027	1.08181	0.1398

	09	1.02826	0.1139	1.05736	0.11028

	10	1.04345	0.112	0.97908	0.12665

	11	0.87012	0.12211	0.97065	0.11379

	12	0.96217	0.13319	1.21053	0.09674

Advocacy	01	0.61039	0.11136	1.00375	0.12929

	02	1.34445	0.11959	1.50981	0.10631

	03	1.17904	0.1215	1.46228	0.11694

	04	1.20342	0.12786	1.2257	0.12075

Opportunity	13	1.09905	0.1241	1.11987	0.14224

	14	1.07315	0.16519	1.0803	0.13446

	16	1.31854	0.11284	1.0526	0.14813

Decision-making	05	0.80304	0.15658	1.21354	0.13519

	15	1.27217	0.15994	1.20523	0.12617


Abbreviations: CFA = Confirmatory factor analysis; PCO = Patient care ownership; EOY = End of year; EFA = Exploratory factor analysis; SE = Standard error. ^a^ All values are significant (p < .001).

For both the EOY 3 and EOY 4 models, the four-factor model had a better fit than the two-factor model across several common indices of model fit, as demonstrated in Appendix E. The four-factor models for both groups had lower AIC and BIC values compared to their respective two-factor models, indicating better model fit. The four-factor model RMSEAs, both below 0.08, indicated fair model fit, and the RMSEAs and SRMRs together indicated acceptable model fit [26]. CFI and TFI values closer to 1 indicate better model fit; values of 0.95 or higher are considered best in most circumstances [25,26]. Although neither of the four-factor models exceeded 0.95 for these metrics, they were closer than their two-factor counterparts.

To assess divergent validity, the inter-factor correlations were examined. In the EOY 3 model, these ranged from 0.31 to 0.88. The Decision-making factor showed higher correlations with the other three factors (0.62 to 0.88) than did the other factors (0.31 to 0.50). The EOY 4 model, the inter-factor correlations were more consistent, ranging from 0.57 to 0.86. Although the correlation between Decision-making and Opportunity was relatively high (0.86), no clear pattern emerged among the other inter-factor correlations in the EOY 4 model.

In summary, four factors – Advocacy, Decision-making, Opportunity, and Responsibility – were found to underpin medical students’ ownership of patient care, as measured by our scale. The final scale organized by factor is included in Appendix D.

## Discussion

A PCO scale designed by Djulbegovic and colleagues for GME learners was revised and evaluated for medical students through a literature review, expert content validation, and cognitive interviewing with medical students. Evidence for two- and four-factor models of PCO was found through exploratory and confirmatory factor analyses, with Advocacy, Decision-making, Opportunity (combined into a single Initiative factor in the two-factor model), and Responsibility identified as subfactors of PCO among medical students. This scale provides a new tool with strong content, response process, and internal structure validity evidence that can be used to measure both affective and task-based elements of PCO among medical students.

Differences between the findings in our study and those of the resident PCO scale written by Djulbegovic et al highlight important differences in the experience of PCO between residents and medical students. The exploratory factor analysis for the Djulbegovic scale revealed three factors that generally correspond to three of our factors: Assertiveness with Advocacy, Serving as the ‘Go-To’ Person with Decision Making, and Diligence with Responsibility. This highlights how several aspects of PCO are preserved across these different groups of learners, even though the exact items that fell into each category differed slightly, as expected across different contexts. We see similar correlates with two of the factors proposed in Wyatt et al.’s analysis: Self-Confidence and Territoriality with Assertiveness/Advocacy, and Accountability with Diligence/Responsibility. However, our fourth factor of Opportunity for PCO is unique and has no correlate in the Djulbegovic scale, suggesting that the opportunity to display PCO is more variable among medical students than among residents. A similar factor in Wyatt’s scale was Team Inclusion, although we view Opportunity as more specific to patient care responsibility. Namely, students must be invited in by patients and their medical team to assume clinical responsibility, which then allows for the development and display of PCO. This finding is in keeping with the framework of legitimate peripheral participation in that students often begin as peripheral participants in patient care but, with graduated responsibility, can become more central members of the medical care teams if they are afforded the chance to do so [16].

We believe the primary differences between the PCO scale presented in this article and the student PCO scale developed by Wyatt et al. reflect differences in our underlying assumptions when adapting the scales and in our approach to gathering validity evidence. Each approach has strengths that can complement each other and spur further investigation. Wyatt et al. directly revised Avey et al.’s psychological ownership scale, whereas we modified the Djulbegovic scale, which had already been adapted for a medical education context from the Avey scale. We chose this approach based on our underlying assumption that medical students can assume graduated responsibility for many of the resident tasks outlined in the Djulbegovic scale. Our scale may serve as an aspirational role framing students in more central roles in patient care as members of the same community of practice. As a result, our scale tends to have more behavioral or task-based items (e.g., “I ensured / challenged / deferred / was / made sure to go back and check) in line with the Djulbegovic adaptations. This is in contrast to Wyatt et al.’s scale, which contains more affective items, such as “I feel / I enjoy / I am / I have a right to,” which are consistent with the original Avey instrument. As we conceptualize PCO as an affective commitment displayed in action [2], we believe both sets of questions can be useful for measuring different aspects of PCO. For example, our work may be more helpful in evaluating a curricular change designed to promote PCO-related behaviors (e.g. LICs which emphasize longitudinal relationships with patients and authentic roles in patient care), while the Wyatt scale would be better-suited for one aimed toward cognitive change (e.g. a case-based learning ethics curriculum focused on complex care decisions). As such, we view the two scales as complementary and hope that future studies can draw on the strengths of each to advance this emerging area of medical education research. Additionally, the similarity in factor solutions provides targets for educational interventions to optimize PCO among students.

In line with our theoretical framework, factor solutions related to task ownership (Responsibility and Decision-making) and their division amongst a team align with the theory of distributed cognition. Within patient care teams, ownership of patient care is shared and must be negotiated among team members, so having a tool to measure students’ roles and contributions will help guide and evaluate the effectiveness of interventions designed to improve PCO in students. Moreover, in accordance with legitimate peripheral participation, our Opportunity factor might be key to affording medical students more central roles in patient care over time, so they can demonstrate the other elements of PCO, such as Responsibility, Advocacy, and Decision-making. The importance of this factor among medical students also provides a key target for intervention among medical educators who wish to optimize development of PCO among students – e.g., affording them opportunities to see patients first, propose treatment plans for their patients before other members of the care team, follow up first on patient laboratory or radiology results, and develop a longitudinal care relationship. Previous studies have explored definitions of PCO and factors affecting its development amongst medical students [23,24,25,26,27], and having a scale with validity evidence to measure it will forward these lines of research by allowing educators to quantify differences in PCO across curricular models, populations, and clinical settings. Findings from our study also affirm students’ ability to assume patient care responsibilities, as evidenced by uniformly high scores on the scale.

Our study has several limitations. The scale was administered to students at a single institution, exclusively to third- and fourth-year medical students, potentially limiting the generalizability of the findings to medical students in other years of training. We intentionally chose this population as several of the scale items involve direct patient care, but future work should further explore the meaning and significance of PCO to students in pre-clinical years of training. Moreover, the AY 20 group was in the clerkship phase of training when clinical curricula were adapted for the COVID-19 pandemic. This may have limited PCO opportunities due to the pause in clinical training activities, decreased operative opportunities from cancelled elective cases, and the shortening of the clinical year (2020–2021 clerkships were one month shorter). However, the scope of medical students’ work expanded in other settings (e.g., delivering care via telehealth), and the psychological impact of belonging to an ‘essential workforce’ may have augmented PCO. Social desirability bias among medical students may contribute to a positive skew in responses regarding core character traits. Survey fatigue could also be reflected in findings as this scale was embedded within a larger global end-of-phase survey. However, the benefit of this approach was the very high response rate. Several items in both the Djulbegovic (item 1) and the adapted (items 2, 3, and 4) could be considered ‘double-barrel’ items, which may be sources of unintended variation in scale interpretation. In future iterations of this scale, certain items could be further revised to minimize this potential source of bias. Furthermore, as the Decision-making factor in our study comprised only two items, we believe this would be a prime area for a future qualitative study to explore which other elements of Decision-making might be appropriate to include in future iterations of this tool. Finally, while qualitative methods were used in scale development, the quantitative aspects of the study cannot speak to the “whys” behind student responses.

We adapted the Djulbegovic scale for use among medical students, aiming to provide medical educators with a tool to quantify PCO. This scale will enable the exploration of the impact of novel curricula on different clinical training models (i.e., LIC vs traditional block rotations) on PCO [28]. The ability to quantify PCO among medical students will also allow future studies to consider how PCO may be correlated with other outcomes of interest, such as academic achievement, wellness, and patient care outcomes. As next steps, we hope to further test this scale among international student populations, as we anticipate that sociocultural factors will influence how PCO is displayed and experienced across contexts. Finally, given the burgeoning field of patient care ownership, developing a deeper understanding of the relative utility of the three available PCO scales across settings, populations, and curricular interventions will allow educators and researchers to further advance this field.

## Previous presentations

An earlier version of this work was presented to the University of Colorado Academy of Medical Educators via virtual posterboard.

## Data Accessibility Statement

The data that support the findings of this study are available from the University of Colorado School of Medicine Office of Assessment, Evaluation, and Outcomes. Restrictions apply to the availability of these data, which were used with permission for this study.

## Additional File

The additional file for this article can be found as follows:

10.5334/pme.1820.s1 Appendices. Appendix A to E.
