# Supplementary material for: Psychometric Analysis of an Adapted Patient Care Ownership Scale for Medical Students
Source: Perspect Med Educ. 2025 Nov 24;14(1):860–70. doi: 10.5334/pme.1820 (PMC12662162; doi:10.5334/pme.1820)
Supplement: Appendices. — Appendix A to E. [file pme-14-1-1820-s1.pdf]

Appendix A: Verbal Script for Medical Student Cognitive Interviews

<u>Questions for cognitive interviews:</u>

Please read the question aloud. Then, select an answer choice. After selecting each answer, I will ask you what the question meant to you and what experiences impacted your choice. After five questions, I will pause and allow you to tell me if any questions were unclear or if you have suggestions.

Responses:

After selecting an answer: *What did the question mean to you?*

After this: *Tell me what experiences you were thinking about when you decided on your answer*.

After each set of 5 questions, *Did you have any questions regarding the questions or suggestions to make any of them clearer?*

<u>Optional:</u>

Shows which questions had been changed from original scale. *Are all of these changes necessary to adapt this scale to medical students?*

Do you feel that these questions are answerable to both your time inpatient and outpatient?

Quantitative coding for each item

Asks for clarification?

Suggested change at any point

Returning to change response?

Retrieval in final month/rotation

Appendix B. Patient Care Ownership Scale Adapted for Medical Students Compared to Original Djulbegovic Scale.

Changes/omissions are bolded. *Justifications are italicized.*

<table>
<tr><th>Item No.</th><th>Djulbegovic Scale Item</th><th>Adapted Scale Item</th></tr>
<tr><td>1</td><td>I was vocal and assertive about my patients’ best treatment/care.</td><td>I was vocal and assertive about my patients’ best treatment/care.</td></tr>
<tr><td colspan="3">Justification: Students and faculty during cognitive interviews believed this reflected an attainable level of advocacy from medical students. We decided to keep the double-barrel aspects, because the nouns (‘vocal’ and ‘assertive’, ‘treatment’ and ‘care’) are similar enough to one another such that maintaining consistency with the Djulbegovic scale outweighed the risk of ambiguous terminology. Further, no concerns were brought forth during student cognitive interviews for this item</td></tr>
<tr><td>2</td><td>I felt comfortable telling the attending what I felt was the right thing to do for my patients, rather than just letting them decide.</td><td>I felt comfortable telling the team and/or attending what I felt was the right thing to do for my patients’ medical conditions, rather than just letting them decide.</td></tr>
<tr><td colspan="3">Justification: 1) Students’ most-immediate supervisor oftentimes are residents, rather than attendings as would be the case for the Djulbegovic GME scale. Thus, the object to whom students are advocating was changed to ‘team and/or attending’. 2) Student cognitive interviews revealed inconsistency in respondents whether ‘the right thing’ meant caring for a patient’s ‘medical conditions’ or ‘social circumstances.’ Accordingly, Djulbegovic item 2 was split into adapted scale items 2 and 3.</td></tr>
<tr><td>3</td><td>New item.</td><td>I felt comfortable telling the team and/or attending what I felt was the right thing to do for my patients’ social circumstances, rather than just letting them decide.</td></tr>
<tr><td colspan="3">Justification discussion above.</td></tr>
<tr><td>4</td><td>I challenged the team as needed if I felt it was in my patients’ best interest, no matter how much push back I got.</td><td>I challenged the team and/or attending’s plan as needed if I felt it was in my patients’ best interest.</td></tr>
<tr><td colspan="3">Justification: This deletion was made after authors discussed i) how medical students are less likely than residents to advocate past several redirections and ii) how the language may contribute to a social desirability bias against rating this item highly.</td></tr>
<tr><td>5</td><td>I frequently deferred to other providers for many aspects of my patients’ care. (reverse)</td><td>I frequently deferred to other providers for many aspects of my patients’ care. (reverse)</td></tr>
<tr><td colspan="3">Justification: We felt that this item is appropriate for the level of a fourth-year medical student to answer affirmatively and chose to include it to gradate responses between medical students based upon their level. While, ‘frequently’ and ‘many’ are subjective terms that may change with level of learner and may be answered similarly based upon students’ perceived expectations, the item becomes more objective when asking about a behavior.</td></tr>
</table>

<table>
<tr><td>6</td><td>I personally made sure to go back and check that all orders were actually carried out.</td><td>I personally made sure to go back and check that all orders were actually carried out.</td></tr>
<tr><td colspan="3">Justification: While this item is aspirational for a third-year medical student, we felt it reasonable to keep this response as is. Moreover, students during semi-structured interviews reflected upon how they felt that this skill grew across their training. However, similar averages across AY 22 EOY 3 and EOY 4 respondents may imply a social desirability bias.</td></tr>
<tr><td>7</td><td>When carrying out my patient’s management plan, I took extra care to make sure that things did not fall through the cracks.</td><td>When carrying out my patient’s management plan, I took extra care to make sure that things did not fall through the cracks.</td></tr>
<tr><td colspan="3">Justification: Please see above. During cognitive interviews, students believed that they could be more detail-oriented in following up on plans because they typically carried fewer patients than residents.</td></tr>
<tr><td>8</td><td>I felt responsible for my patients’ care, even after my shift ended.</td><td>I felt responsible for my patients’ care, even after my shift ended.</td></tr>
<tr><td colspan="3">Justification: This felt appropriate for all levels of learner. Students often cited a sense of anxiety when leaving their inpatient rotations, hoping they would do well overnight during cognitive interviews.</td></tr>
<tr><td>9</td><td>I was the "go-to" person for knowledge about my patients.</td><td>I was the "go-to" person for knowledge about my patients.</td></tr>
<tr><td colspan="3">Justification: Similar to item 11, we included more aspirational items for students in hopes of achieving a better gradiation of responses.</td></tr>
<tr><td>10</td><td>I made sure that the nursing staff was updated with the day’s plan.</td><td>I made sure that non-physician health care providers were updated with the plan.</td></tr>
<tr><td colspan="3">Justification:
i) ‘Non-physician health care providers’ was felt to be more inclusive than ‘nursing staff’ to expert reviewers. It was also discussed among authors that the primary outpatient team with whom medical students work often involves medical assistants, social workers, and pharmacists to a sometimes greater degree than the inpatient setting.
ii) ‘Day’s plan’ was adapted to ‘the plan’ to focus less on inpatient settings. This theme arose from student interviews.</td></tr>
<tr><td>11</td><td>I was proactive in checking up on my patients, rather than being called with questions or concerns.</td><td>I was proactive in checking up on my patients, rather than waiting to be called with questions or concerns.</td></tr>
<tr><td colspan="3">Justification: After external expert revision, a change in semantics was made to clarify that ‘to be called’ meant ‘brought to one’s attention’ rather than ‘to receive a phone call as the provider’, a role to which early clinical students do not often have access.</td></tr>
<tr><td>12</td><td>I ensured good continuity of care even when I was absent from the service.</td><td>I ensured good continuity of care even when I was not present.</td></tr>
<tr><td colspan="3">Justification: Revised after initial author agreement that ‘absence from the service’ implied inpatient practice.</td></tr>
<tr><td>13</td><td>I was given the opportunity to make decisions independently about my patients’ care.</td><td>I was given the opportunity to make decisions independently about my patients’ care.</td></tr>
</table>

<table>
<tr><td colspan="3">Justification: This was another item that seemed aspirational for medical students, but this related to their desire to at least propose initial management plans and decisions for their patients.</td></tr>
<tr><td>14</td><td>I felt that my attending(s) micromanaged me (reverse)</td><td>I felt I was not given enough autonomy in patient care. (reverse)</td></tr>
<tr><td colspan="3">Justification: Expert discussion and student interviews were concordant that ‘micromanaged’ was both i) more applicable to residents who tend to be more directly managed by attendings and ii) emotionally-charged enough of a word that it is vulnerable to social desirability bias in responding with either direction based on emotions related to experiences with attendings rather than the sentiment of autonomy. Students rarely perceived lack of autonomy as a negative when considering their experience in the principal clinical year. Rather, they found more oversight to imply safer patient care but started to wish for more autonomy when thinking about experiences as a fourth-year student (e.g., hoping to speak directly with consultants and summarize recommendations for the team).</td></tr>
<tr><td>15</td><td>I felt comfortable making decisions independently about my patients’ care.</td><td>I felt comfortable making decisions independently about my patients’ care.</td></tr>
<tr><td colspan="3">Justification: Students viewed this item as independent thoughts rather than independent management, which felt appropriate for their development as a learner. Some also perceived it as being related to the psychological safety of their team which may serve as a confounder.</td></tr>
<tr><td>16</td><td>New item</td><td>I felt I had sufficient opportunity to take ownership of my patients’ care.</td></tr>
<tr><td colspan="3">Justification: Student reviewers felt that the perception of opportunity for care ownership is distinct from other survey questions. Nearly uniformly, interviewees independently discussed how this sentiment differed from residents for whom the opportunity for care is often an implied responsibility.</td></tr>
<tr><td>17</td><td>I felt a strong sense of ownership of my patients’ care.</td><td>I felt a strong sense of ownership of my patients’ care.</td></tr>
<tr><td colspan="3">Justification: We felt no further modification of this item was necessary, as with other items exploring cognitive aspect of PCO.</td></tr>
</table>

Appendix C: Items means and standard deviations.

	AY 21 EOY 3 N = 176		AY 22 EOY 3 N = 167		AY 22 EOY 4 N = 151	
	Mean	SD	Mean	SD	Mean	SD
Item 1	5.42	1.06	6.31	1.21	6.23	1.40
Item 2	5.19	1.35	6.26	1.51	5.97	1.63
Item 3	5.34	1.24	6.24	1.46	6.07	1.62
Item 4	4.51	1.55	5.12	1.98	5.22	1.78
Item 5	4.10	1.54	4.72	1.95	5.01	1.93
Item 6	4.91	1.47	5.67	1.92	5.79	1.80
Item 7	5.68	0.99	6.70	1.18	6.59	1.27
Item 8	5.50	1.49	6.16	1.85	6.23	1.71
Item 9	5.32	1.20	6.32	1.55	6.35	1.39
Item 10	5.28	1.18	6.07	1.52	6.20	1.44
Item 11	5.84	1.06	6.80	1.14	6.64	1.21
Item 12	5.27	1.22	6.16	1.50	5.96	1.62
Item 13	5.38	1.21	6.29	1.55	6.49	1.37
Item 14	4.53	1.62	5.50	1.94	5.81	1.76
Item 15	4.94	1.24	5.49	1.74	5.94	1.54
Item 16	5.14	1.36	6.08	1.59	6.36	1.47
Item 17	5.49	1.32	6.49	1.40	6.49	1.34

Appendix D. Final Medical Student Patient Care Ownership Scale Organized by Factor

Scale numbers included.

Advocacy

1. I was vocal and assertive about my patients' best treatment/care.
2. I felt comfortable telling the team and/or attending what I felt was the right thing to do for my patients' medical conditions, rather than just letting them decide.
3. I felt comfortable telling the team and/or attending what I felt was the right thing to do for my patients' social circumstances, rather than just letting them decide.
4. I challenged the team and/or attending's plan as needed if I felt it was in my patients' best interest.

Responsibility

6. I personally made sure to go back and check that all orders were actually carried out.
7. When carrying out my patient's management plan, I took extra care to make sure that things did not fall through the cracks.
8. I felt responsible for my patients' care, even after my shift ended.
9. I was the "go-to" person for knowledge about my patients.
10. I made sure that non-physician health care providers were updated with the plan.
11. I was proactive in checking up on my patients, rather than waiting to be called with questions or concerns.
12. I ensured good continuity of care even when I was not present.

Decision-making

5. I frequently **deferred** to other providers for many aspects of my patients' care. *(reverse)*
15. I felt comfortable making decisions independently about my patients' care.

Opportunity

13. I was given the opportunity to make decisions independently about my patients' care.
14. I felt I was **not** given enough autonomy in patient care. *(reverse)*
16. I felt I had sufficient opportunity to take ownership of my patients' care.

Omitted due to cross-loading among multiple factor solutions

17. I felt a strong sense of ownership of my patients' care.

Appendix E. Comparison of Two and Four Factor Solutions across Indices of Model Fit.

Factor Model and Year	AIC	BIC	CFI	TLI	RMSEA	SRMR
Two-factor, EOY3	351.583	454.477	0.742	0.699	0.103	0.105
Two factor, EOY4	322.960	421.868	0.803	0.7704	0.101	0.098
Four factor, EOY3	266.502	384.986	0.869	0.840	0.075	0.081
Four factor, EOY4	244.726	358.621	0.910	0.889	0.070	0.075

Abbreviations: EOY = End of Year; AIC = Akaike information criterion; BIC = Bayesian information criterion; CFI = Comparative Fit Index; TLI = Tucker-Lewis Index; RMSEA = Root Mean Square Error of Approximation; SRMR = Standardized Root Mean Squared Residual
